# PRELIMINARY EFFICACY OF CARFREEME™-DEMENTIA: A DRIVING RETIREMENT PROGRAM FOR FAMILIES IN THE US

**DOI:** 10.1093/geroni/igad104.1354

**Published:** 2023-12-21

**Authors:** Colleen Peterson, Stephanie N Ingvalson, Robyn Birkeland, Katie Louwagie, Maya Koffski, Joseph Gaugler

**Affiliations:** University of Michigan, Ann Arbor, Michigan, United States; University of Minnesota, Minneapolis, Minnesota, United States; University of Minnesota, Minneapolis, Minnesota, United States; University of Minnesota, Minneapolis, Minnesota, United States; University of Minnesota, Minneapolis, Minnesota, United States; University of Minnesota, Minneapolis, Minnesota, United States

## Abstract

Driving retirement can be a disruptive decision for people with Alzheimer’s disease and related dementias (AD/ADRD) and their families. Retiring drivers with AD/ADRD may lose their sense of independence and connection to community. Family members can experience relationship stress convincing persons with dementia to stop driving and feel strained taking on transportation responsibilities. CarFreeMe™-Dementia (CFM™-D) is an individualized psychosocial driving retirement program for families who are planning for, implementing, or adjusting to driving retirement. A small feasibility study of the program in the U.S. indicated that participants found it acceptable and useful for navigating driving retirement. Phase 2 of this study, enrolling 17 care partners, 16 retiring drivers, and 17 dyads, evaluated the preliminary efficacy of CFM™-D to provide emotional support and increase driving retirement readiness. Data from baseline, 3-, and 6-month surveys and an exit semi-structured interview were collected. Interviews confirmed that CFM™-D provided emotional support and practical preparation for driving retirement. Chi-square analyses comparing baseline to 6-month follow-up data showed fewer caregivers reached thresholds for concern on scales of strain (p = .019) and isolation (p = .001). Retiring drivers reported reductions in loneliness at 6-months (p = .017) and improved Assessment of Readiness for Mobility Transition scores at 3- and 6-month follow-ups (p = .003, p = .014, respectively). Findings have informed intervention delivery improvements for a future randomized trial evaluating whether CFM™-D is an efficacious tool for addressing the psychosocial complexities of driving retirement amongst people with AD/ADRD and their family.

